# Perilla Seed Oil: A Review of Health Effects, Encapsulation Strategies and Applications in Food

**DOI:** 10.3390/foods13223615

**Published:** 2024-11-13

**Authors:** Min Li, Nanjie Jiang, Guangqi Guo, Shuaijun Lu, Ziliang Li, Yujie Mu, Xiaoyang Xia, Zhenxia Xu, Yong Hu, Xia Xiang

**Affiliations:** 1 Key Laboratory of Fermentation Engineering (Ministry of Education), Hubei Key Laboratory of Industrial Microbiology, National “111” Center for Cellular Regulation and Molecular Pharmaceutics, Hubei Research Center of Food Fermentation Engineering and Technology, Cooperative Innovation Center of Industrial Fermentation (Ministry of Education & Hubei Province), Hubei University of Technology, Wuhan 430068, China; lm2116309327@163.com; 2 Oil Crops Research Institute of Chinese Academy of Agricultural Sciences, Oil Crops and Lipids Process Technology National & Local Joint Engineering Laboratory, Key Laboratory of Oilseeds Processing, Ministry of Agriculture and Rural Affairs, Hubei Key Laboratory of Lipid Chemistry and Nutrition, Wuhan 430062, China; 82101235565@caas.cn (N.J.); 13296554580@163.com (G.G.); lu_shuai_jun@163.com (S.L.); silverssa@outlook.com (Z.L.); m15628003749@163.com (Y.M.); xia_xiaoyang@126.com (X.X.); xuzhenxia1993_2@163.com (Z.X.); 3 Key Laboratory for Green Chemical Process of Ministry of Education, Hubei Key Laboratory for Novel Reactor and Green Chemistry Technology, School of Chemical Engineering and Pharmacy, Wuhan Institute of Technology, Wuhan 430073, China; 4 School of Pharmacy, Xinxiang Medical University, Xinxiang 453003, China

**Keywords:** perilla seed oil, health effects, intestinal microbiota, encapsulation

## Abstract

Perilla (*Perilla frutescens* L.) is an annual herbaceous plant whose seed oil is rich in unsaturated fatty acids such as alpha-linolenic acid (ALA). This oil exhibits various health benefits, including antioxidant, anti-inflammatory, lipid-lowering, hypoglycemic, neuroprotective and immunomodulatory activities. In addition, incorporating perilla oil into a diet can effectively increase the abundance of beneficial bacteria in the gut microbiota. However, perilla oil is prone to oxidation, which reduces its nutritional value and lowers its bioavailability. To address these issues, encapsulation technologies such as emulsions, oleogels, liposomes and microcapsules have been employed, showing promising results. Nonetheless, further research is needed to fully elucidate the underlying mechanisms of perilla seed oil’s health effects, validate its benefits through large-scale human clinical trials and optimize encapsulation techniques. Future investigations should also explore the synergistic effects of combining perilla seed oil with other functional components and its role in modulating gut microbiota to achieve comprehensive health benefits.

## 1. Introduction

*Perilla frutescens* (L.), belonging to the *Lamiaceae* family, is an annual herb commonly known as perilla, shiso or beefsteak plant [1,2]. It is a traditional plant used for both culinary and medicinal purposes, predominantly found in East Asia, North America and Europe [3]. As a traditional Chinese medicinal herb, perilla has been included in several authoritative pharmacopeias, such as the Pharmacopoeia of the People’s Republic of China. Perilla seed oil (PSO) constitutes approximately 40% to 50% of the perilla seed content [4]. Market research data from 2021 indicate that the global market size for PSO reached USD 874.9 million, with projections to expand to USD 2.4156 billion by 2031, reflecting a compound annual growth rate of 10.6% during the forecast period. These statistics highlight the promising market prospects for PSO.

**Figure 1:**
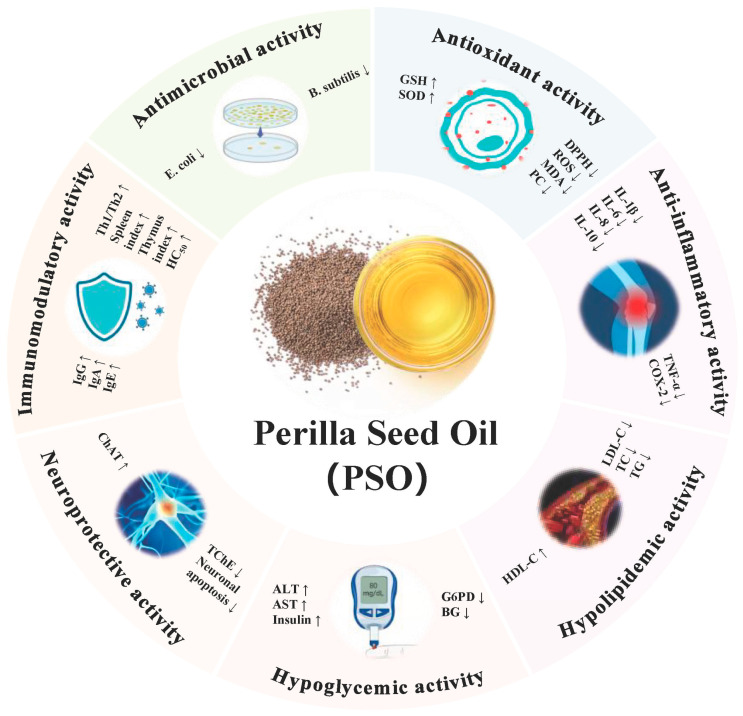
The bioactivity of PSO (perilla seed oil) includes its effects in antimicrobial, antioxidant, anti-inflammatory, hypolipidemic, hypoglycemic, neuroprotective and immunomodulatory activities. Abbreviations: GSH, glutathione; SOD, superoxide dismutase; DPPH, 2,2-diphenyl-1-picrylhydrazyl; ROS, reactive oxygen species; MDA, malondialdehyde; LPO, lipid peroxidation; TNF-α, tumor necrosis factor-alpha; COX-2, cyclooxygenase-2; IL-1β, interleukin-1 beta; IL-6, interleukin-6; IL-10, interleukin-10; LDL-C, low-density lipoprotein cholesterol; TC, total cholesterol; TG, triglycerides; HDL-C, high-density lipoprotein cholesterol; ALT, alanine aminotransferase; AST, aspartate aminotransferase; G6PD, glucose-6-phosphate dehydrogenase; BG, blood glucose; ChAT, choline acetyltransferase; TChE, total cholinesterase; IgG, immunoglobulin G; IgA, immunoglobulin A; IgE, immunoglobulin E.

PSO is considered a high-quality nutritional edible oil. The fatty acid composition of perilla seed oil is relatively complex, with polyunsaturated fatty acids (PUFAs) making up 70.7% to 83.4% of the total, including palmitic acid (5.1–7.0%), stearic acid (1.1–2.3%), oleic acid (9.6–20.8%), linoleic acid (9.5–14.4%) and α-linolenic acid (58.8–70.9%). This oil ranks among the highest in α-linolenic acid content compared to other plant seed oils [5,6]. The proportions of these components may vary depending on the cultivar, extraction and measurement methods. Additionally, perilla seed oil is rich in phytosterols, including β-amyrin, β-sitosterol, campesterol and stigmasterol, with the highest cultivar showing a total sterol content of 402.66 ± 25.36 mg/100 g. β-sitosterol is the predominant phytosterol at 385.44 ± 25.09 mg/100 g, constituting the majority of total sterols, followed by campesterol (10.95 ± 0.24 mg/100 g) and stigmasterol (5.72 ± 0.02 mg/100 g). The β-sitosterol content in PSO is significantly higher than in other vegetable oils, such as corn oil (266.3 mg/100 g) and flaxseed oil (237.5 mg/100 g) [7]. Furthermore, perilla oil is rich in tocopherols (64.115–70.2 mg/100 g), with γ-tocopherol comprising 56.899–65.0 mg/100 g, accounting for 88.7–92.59% of the total tocopherol content; α-tocopherol is present at 3.0–5.165 mg/100 g, δ-tocopherol at 1.7–2.059 mg/100 g and β-tocopherol at 0.4 mg/100 g [8,9]. Research has demonstrated that PSO exhibits multiple biological activities, including antioxidant, anti-inflammatory, lipid-lowering, blood glucose-lowering, neuroprotective and immunomodulatory effects (Table 1 and Figure 1). Furthermore, these bioactivities are closely linked to its capacity to modulate gut microbiota. However, due to the presence of unsaturated double bonds and diallyl methylene groups in PSO, and its non-water-soluble nature, it is prone to oxidation, isomerization and polymerization under environmental stress, leading to low bioavailability [10]. To enhance the stability and bioavailability of PSO, various encapsulation technologies such as emulsions, oleogels, liposomes and microcapsules are currently being extensively explored.

This review systematically summarizes recent research progress on PSO, with a particular focus on its biological activities, its health benefits through the modulation of gut microbiota and the latest developments in encapsulation technologies and their applications. The aim is to provide a theoretical foundation and scientific guidance for the development of PSO and related nutritional and health products.

## 2. Health Benefits of PSO

### 2.1. Antioxidant Activity

Antioxidant activity is one of the primary health benefits of PSO, and it has been extensively studied. Research indicates that PSO exhibits significant antioxidant capacity in in vitro chemical antioxidant assays, with half-maximal inhibitory concentrations (IC_50_) for superoxide anion (O^2−^) and nitrite (NaNO_2_^−^) scavenging being 2.49 mg/mL and 2.51 mg/mL, respectively, which has good antioxidant activity [11]. Moreover, the antioxidant activity of PSO is significantly influenced by its processing method. Studies have found that infrared pre-treatment enhances the DPPH radical scavenging ability of PSO to 73.14%, representing an increase of 22.17% compared to the untreated control group’s 52.07%. Simultaneously, the total phenolic content of PSO shows an increasing trend following infrared pre-treatment, with the highest concentration of total phenolics, 13.65 mg GAE/100 g [12], observed after treatment at 120 °C for 10 min. The enhanced antioxidant activity of infrared-treated PSO is likely attributed to the increased polyphenol content [30], the main polyphenols in PSO include vanillic acid (68.6 ng/g), ferulic acid (55.7 ng/g), apigenin (43.3 ng/g) and caffeic acid (32.8 ng/g) [31].

To further substantiate the antioxidant activity of PSO, researchers employed cellular models for verification. PSO was found to significantly reduce ROS levels in mouse embryonic fibroblasts treated with H_2_O_2_ [13]. Additionally, PSO at a concentration of 400 µg/mL markedly decreased oxidative stress in A549 lung adenocarcinoma cells induced by TNF-α. In in vivo antioxidant activity assays, PSO exhibited significant antioxidant effects in a mouse model of ethanol-induced oxidative damage. These effects were demonstrated by reducing malondialdehyde (MDA) levels in the liver by 46.08%, lowering protein carbonyl (PC) levels, increasing glutathione (GSH) content and enhancing the activity of superoxide dismutase (SOD) in serum [14,15]. The findings suggest that PSO has substantial potential as an antioxidant, further supporting its application value in the development of functional foods and pharmaceuticals. Another study showed that at a PSO dose of 0.815 mL/kg, PSO significantly increased serum SOD levels in rats, reaching 230.21 ± 45.16 U/mL, while significantly reducing the MDA content to 3.75 ± 0.68 nmol/mL. This indicates that PSO can effectively enhance the learning and memory abilities as well as the physical strength of aging rats [32]. Some studies have explored the role of PSO in relieving cold stress. The experimental results showed that the SOD activity was 1.15 times higher and the MDA level was 11.26% lower than that of the model group after 9 days of feeding. The strong antioxidant capacity of PSO was further confirmed [33]. 

### 2.2. Anti-Inflammatory Activity

When exposed to exogenous stimuli, the body defends against pathogen infection or tissue damage through an inflammatory response [34]. However, excessive inflammatory responses indicate dysregulated inflammation, often leading to various diseases. Studies have demonstrated that PSO exhibits significant anti-inflammatory activity. In vitro cell experiments showed that PSO, within a concentration range of 25 to 400 μg/mL, significantly reduced the mRNA expression levels of interleukin-1β (IL-1β), interleukin-6 (IL-6), interleukin-8 (IL-8), tumor necrosis factor-α (TNF-α) and cyclooxygenase-2 (COX-2) in TNF-α-induced A549 cells. Moreover, leukotriene B4 (LTB4), a critical inflammatory mediator, plays a crucial role in inflammatory diseases such as asthma. PSO was able to inhibit LTB4 release from polymorphonuclear leukocytes induced by the calcium ionophore A23187, achieving an inhibition rate of 57.9% at a concentration of 0.4 g/L. This suggests that PSO may exert anti-inflammatory and anti-asthmatic effects by inhibiting the leukotriene biosynthesis pathway [16].

Additionally, PSO significantly inhibited ethyl phenylpropiolate (EPP)-induced ear swelling in rats. At doses of 2.5 and 5 mL/kg, PSO showed inhibition rates of 16.8% and 18.0%, respectively, against acetic acid-induced writhing responses in mice, and inhibition rates of 59.3% and 65.7%, respectively, against EPP-induced ear edema in rats [17]. Furthermore, in a high-fat diet mouse model, PSO demonstrated protective effects against dextran sulfate sodium-induced colitis, as evidenced by significant reductions in the levels of TNF-α, IL-6 and IL-1β in both serum and colon tissue, consistent with in vitro cell experiment results. The research also found that replacing part of the dietary fat in mice with PSO significantly reduced the levels of pro-inflammatory cytokines (IL-1β, TNF-α and IL-10) secreted by lung tissue cells under lipopolysaccharide (LPS) stimulation, decreasing by 62.9%, 47.3% and 62.7%, respectively [35]. PSO has potential application value in modulating inflammatory responses and alleviating inflammation-related diseases. However, the specific molecular mechanisms underlying its effects remain not fully elucidated. Future research should focus on uncovering the precise molecular mechanisms of PSO’s anti-inflammatory action to clarify its role in signaling pathways.

### 2.3. Hypolipidemic Activity

Long-term high-fat diets (HFD) can lead to a range of diseases and symptoms, including lipid metabolism disorders, dyslipidemia, obesity, diabetes, hyperlipidemia and non-alcoholic fatty liver disease (NAFLD) [36]. Various functional foods containing natural bioactive compounds have been shown to ameliorate hyperlipidemia with minimal side effects [37]. PSO has demonstrated notable hypolipidemic activity, as it can reduce the levels of total cholesterol (TC), triglycerides (TG), low-density lipoprotein cholesterol (LDL-C) and MDA in hyperlipidemic mouse models, while simultaneously increasing high-density lipoprotein cholesterol (HDL-C), SOD and catalase (CAT) levels [18,19]. 

Moreover, PSO can be mixed with other oils to achieve enhanced lipid-lowering effects. Both linoleic acid and α-linolenic acid have been shown to exert lipid-lowering effects. Safflower seed oil, which is rich in linoleic acid with a content ranging from 73% to 85%, and PSO, which contains over 90% unsaturated fatty acids, particularly high levels of α-linolenic acid, can be combined for better regulation of blood lipid levels [20]. Studies have found that different ratios of safflower seed oil to PSO significantly impact the blood lipid levels of mice and rats. Specifically, at ratios of 2.59:1 and 5.42:1, the levels of TC, TG and LDL-C in the serum of mice decreased by 16.8% and 12.4%, 16.8% and 14.3%, 16.9% and 3.9%, respectively, while HDL-C increased by 42.8% and 28.3%. In rats, ratios of safflower seed oil to PSO at 2.59:1 and 0.47:1 also resulted in significant reductions in TC, TG and LDL-C levels by 39.8% and 33.6%, 50.9% and 33.3%, 31.3% and 18.8%, respectively, with HDL-C increasing by 37.2% and 10.9% [38]. These changes in hepatic lipid levels were consistent with the observed serum lipid changes. Yu et al. [39] investigated the effects of PSO combined with safflower seed oil in equal proportions on blood lipid levels in hyperlipidemic mice. The results indicated that the low-dose, high-dose and PSO soft capsule groups reduced TC by approximately 21.7%, 24.4% and 14.1%, and TG by approximately 43.4%, 48.7% and 28.9%, respectively. In terms of the HDL-C/TC ratio, increases of approximately 70%, 77.5% and 40% were observed, indicating that the combination of PSO and safflower seed oil can more effectively improve blood lipid levels [40]. Additionally, the combination of PSO with flaxseed oil also exhibited significant hypolipidemic activity, with doses ranging from 250–1500 mg/kg resulting in a marked reduction in TC and TG [39].

### 2.4. Hypoglycemic Activity

PSO also has potential hypoglycemic effects. Research conducted by wang et al. [21] reported that PSO significantly elevated the levels of albuminous aminotransferase (ALT) and aspartate aminotransferase (AST), while inhibiting glucose, glucose-6-phosphate dehydrogenase, TG and TC levels, suggesting that PSO may serve as a potential therapeutic agent for diabetes. Although there were no significant differences in blood glucose and insulin levels between the PSO intervention group and the diabetic model group, the TG levels in the group receiving 0.67 g/kg BW/day PSO were significantly reduced by 19.27%. Moreover, PSO supplementation alleviated histopathological changes, including hepatic steatosis and adipocyte hypertrophy. These findings indicate that PSO indirectly promotes glucose metabolism by reducing TG levels and improving liver and adipose tissue health [21]. Furthermore, the study demonstrated that PSO effectively improved hyperglycemia in a type 2 diabetic KKAy mouse model. Specifically, after 28 days of PSO intervention, the blood glucose levels of all mice decreased to below 7 mM, reaching normal levels. Additionally, insulin levels in the PSO-treated group were approximately 1.33 times higher than those in the model group, indicating that PSO exhibited significant hypoglycemic effects in the type 2 diabetic KKAy mouse model [22].

### 2.5. Neuroprotective Activity

PSO has demonstrated significant neuroprotective activity. In the study by wu et al. [23], a chronic cerebral hypoperfusion model was established in rats using the bilateral common carotid artery occlusion method, and cognitive function was assessed via the Morris water maze test. PSO, administered at doses ranging from 0.2 mL/(100 g) to 0.8 mL/(100 g), shortened the escape latency by 36.5% to 45.0%, reduced total cholinesterase (TChE) activity by 6.4% to 19.5% and increased choline acetyltransferase (ChAT) activity by 18.7% to 40.3%. These findings suggest that PSO effectively mitigates cognitive impairment induced by chronic cerebral hypoperfusion, potentially through the inhibition of TChE activity and the enhancement of ChAT activity. Additionally, at doses of 1.1 g/(kg·d) to 11 g/(kg·d), PSO continued to reduce the escape latency by 22.6% to 31.7% [23]. Specifically, escape latency times for young rats decreased by approximately 22.7%, 28.8% and 31.6% at these dosage levels, respectively. Regarding the number of platform crossings, the medium-dose group (2.2 g/(kg·d)) exhibited an increase of approximately 91%, indicating a significant enhancement in spatial learning and memory capabilities in weanling rats. The neuroprotective effects of PSO may be attributed to its ability to reduce oxidative stress. This was evidenced by increased SOD activity and decreased MDA levels, which may play a role in the observed improvements in cognitive function [24]. In Apolipoprotein E (ApoE) KO mice fed with PSO, the incidence of dark neurons was significantly reduced by approximately 35%, and neuronal apoptosis in the brain was reduced by around 37%. These results indicate that PSO may help prevent brain damage associated with hypercholesterolemia by alleviating oxidative stress, inflammation and neuronal apoptosis [25].

### 2.6. Immunoregulatory Activity

Adding 0.6% PSO to the diet can enhance the immune performance of healthy layer chicks, particularly by increasing IgG levels. The ALA rich in PSO may strengthen the immune system by regulating molecular expression on immune cell membranes and increasing immunoglobulin levels in the blood [26]. Furthermore, Song et al. demonstrated that perilla seed extract had a more potent effect on enhancing broiler chicken immunity compared to chlortetracycline treatment, as assessed through various survival and immune indices [41]. However, another study found that adding 5% PSO to the diet reduced OVA-specific IgG1 antibody levels by 22.6%, demonstrating certain anti-allergic potential in OVA-sensitized and challenged mouse models. At the same time, it increased non-specific IgE levels by 28.9%, which may activate mast cells and lead to the rapid release of inflammatory mediators, potentially triggering allergic reactions. Additionally, PSO lowered non-specific IgA levels by 17.3%, which might be beneficial for allergic asthma inflammation [27]. Moreover, PSO did not significantly alter the Th1/Th2 antibody balance. These findings suggest that while PSO plays a role in modulating immune responses, its impact on increasing total IgE levels indicates a complex immunomodulatory effect.

In animal models, PSO administered at doses ranging from 50 to 200 mg/kg enhanced spleen and thymus indices in rats. A dose of 200 mg/kg increased the spleen index by 20.75%, the thymus index by 27.54%, the phagocytic rate by 92.94% and the phagocytic index by 57.14%. The half hemolysis value (HC_50_) also improved by approximately 50.30% [28]. These findings highlight that PSO can effectively counteract exercise-induced declines in immune function, particularly by enhancing macrophage phagocytic activity and increasing the indices of immune organs, thereby strengthening the humoral immune function in exercising rats.

### 2.7. Other Bioactivities

The antimicrobial activity of oils is gaining increasing attention, and PSO has demonstrated notable antibacterial properties. The inhibition rates of PSO against Escherichia coli and Bacillus subtilis range from 53.3% to 101.7% and 108.3% to 136.7%, respectively, which are significantly higher than those of soybean oil, with inhibition rates of 50.0% to 88.3% and 50.0% to 68.3%, respectively [29].

Additionally, PSO has anti-thrombotic and anti-atherosclerotic properties. Jang [42] and colleagues reported that PSO can inhibit platelet aggregation by blocking the production of thromboxane, thus delaying thrombus formation following oxidative arterial wall injury. At a dose of 2 mL·kg⁻¹, the effect of PSO in preventing vascular obstruction was comparable to that of aspirin (30 mg·kg⁻¹) [42]. Researchers further studied the anti-atherosclerotic effects of PSO, finding that it not only alleviated hypercholesterolemia and atherosclerosis but also reduced fat accumulation and lipid peroxidation reactions in the liver and kidney tissues. This suggests that PSO, by regulating lipid metabolism, can prevent the formation of atherosclerosis and fatty liver, making it an ideal oil for improving diet-induced metabolic syndrome [43]. In cardiovascular function studies, the α-linolenic acid found abundantly in PSO can be converted into eicosapentaenoic acid and docosahexaenoic acid in the body, which are essential fatty acids beneficial to the cardiovascular system. These acids can lower TG levels in the blood, reduce inflammation, lower blood pressure and improve blood circulation. Moreover, PSO is rich in various antioxidants, such as polyphenolic compounds, vitamin E and carotenoids, which can combat free radical damage and protect cardiovascular tissues from oxidative stress, thereby reducing the risk of atherosclerosis and thrombus formation [41]. 

Another study explored the role of PSO in alleviating cold stress. Under cold stress conditions, mice that ingested PSO had a body temperature 0.5 °C higher than the model group, approaching the normal temperature of the control group (NC group). Experimental results showed that after nine days of feeding, the body weight of mice in the PSO group tended to normalize, the white blood cell count was 1.43 times higher than that of the model group, the SOD activity was 1.15 times higher and the MDA level was 11.26% lower than in the model group. These findings suggest that PSO can effectively alleviate the impact of cold stress on mice [33].

## 3. Gut Microbiome

The gut microbiota is closely associated with host health, comprising a vast and diverse community that plays a crucial role in maintaining intestinal homeostasis through mechanisms such as biological antagonism, immune regulation and metabolic modulation [44]. These interactions ensure the proper functioning of the gastrointestinal system. Recent studies have demonstrated that dysbiosis of the gut microbiota is linked to various human diseases. As a bridge between diet and the host, the composition of the gut microbiota can be modulated by altering dietary patterns, which in turn affects metabolic products and nutrient metabolism [14]. The dominant phyla in the human gut are Firmicutes and Bacteroidetes, which together constitute more than 90% of the microbial population. Other prominent phyla include *Proteobacteria*, *Verrucomicrobia*, *Actinobacteria* and *Fusobacteria* [45].

A growing body of research has investigated the impact of edible oils on the gut microbiota. Flaxseed oil, camellia oil, walnut oil and olive oil have all been shown to increase the abundance of *Firmicutes*. Both flaxseed oil and olive oil have been observed to reduce the abundance of Bacteroidetes, while camellia oil regulates the gut microbiota by increasing the *Firmicutes*-to-*Bacteroidetes* (F/B) ratio. Additionally, studies on flaxseed oil, olive oil and walnut oil consistently report a decrease in the relative proportion of *Proteobacteria*. These overlapping microbial changes suggest that, while different edible oils may modulate gut microbiota with varying degrees of emphasis on specific aspects, they share commonalities in promoting beneficial bacteria such as *Firmicutes* and reducing harmful bacteria such as *Proteobacteria* [46,47,48,49,50].

Dietary supplementation with PSO has been linked to the promotion of intestinal health, supporting a higher biodiversity and greater structure of gut microbiota. PSO exerts direct effects on the composition and structure of the gut microbiome, fostering the growth of beneficial bacteria while inhibiting the proliferation of harmful species. Notably, PSO consumption has been associated with a significant reduction in the abundance of *Blautia*, accompanied by the proliferation of *Lactobacillus*, as observed in diabetic KKAy mice models. *Blautia* is a Gram-positive, anaerobic bacterium belonging to the family *Lachnospiraceae*, which has been implicated in the development of disturbances in glucose metabolism. In contrast, *Lactobacillus*, typically regarded as a beneficial bacterium, is well known for its ability to convert sugars into lactic acid, contributing to gut health [21]. Additionally, PSO treatment in type-2 diabetic KKAy mice has been shown to significantly reduce the abundance of *Aerococcus* while promoting the richness of *Alloprevotella* and *Akkermansia* in the gut. *Aerococcus* is a pathogenic bacterium associated with infections in both humans and animals. In contrast, *Alloprevotella* is often negatively correlated with disease and produces butyric acid, a short-chain fatty acid (SCFA) known to ameliorate the effects of type 2 diabetes. *Akkermansia* is well-recognized for its role in SCFA production, which contributes to improved gut permeability, enhanced metabolic function and maintenance of intestinal barrier integrity. Furthermore, *Akkermansia* stimulates mucus secretion, fortifies the intestinal barrier, reduces circulating LPS levels and alleviates inflammation [22]. The F/B ratio is a key indicator of host health, with an increased F/B ratio commonly observed in HFD-fed mice, marking the gut dysbiosis associated with obesity. Aldamarany et al. (2023) demonstrated that dietary PSO treatment reduced the relative abundance of Firmicutes compared to the HFD group, while increasing the abundance of Bacteroides, thereby restoring the F/B ratio to levels similar to the control group [51]. In the process of PSO gavage, the enhancement of bile secretion may significantly influence the gut microbiota. Bile acids play a critical role in the digestion and absorption of fats. Following the administration of the oil, bile acids are released to facilitate the solubilization of fats and are subsequently metabolized by the gut microbiota, resulting in the production of important secondary metabolites such as SCFAs, trimethylamine and secondary bile acids. These metabolites are significant in regulating the fat–bile–gut axis. Additionally, ALA, a key component of PSO, may exert antimicrobial effects by inhibiting bacterial fatty acid synthesis, which is crucial for bacterial survival and proliferation [21].

## 4. Encapsulation Strategies and Their Applications in Food

**Figure 2:**
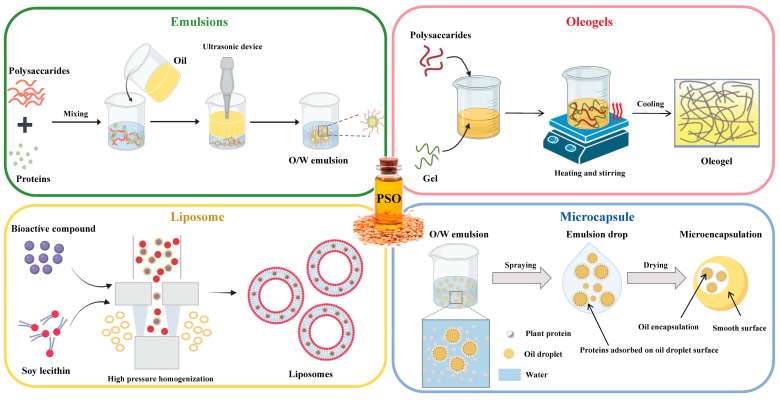
Encapsulation strategies of PSO, including emulsions, oleogels, liposomes and microcapsules.

Due to the presence of a highly unsaturated double bond structure and bis-allyl methylene groups, PSO is prone to oxidation, isomerization and polymerization under external environmental stress, resulting in its low bioavailability. This inherent instability is closely associated with the hydrophobic characteristics of PSO [10]. These properties significantly limit the application of PSO during processing, storage and transportation, thereby diminishing its bioactivity and bioavailability. To address these issues, encapsulation techniques have been proven effective in retarding oxidative processes, thereby enhancing the stability of unsaturated lipids. Various encapsulation strategies have been developed to improve the bioavailability of PSO, including emulsions, oleogels, liposomes and microcapsules (Figure 2 and Table 2). These technological approaches help to overcome the stability challenges associated with the application of PSO.

### 4.1. Emulsions

Emulsions play a crucial role in enhancing food properties and improving the water solubility and bioavailability of bioactive food components [72].The physicochemical properties of emulsions are significantly influenced by the choice of emulsifiers, oil phase ratios and other stabilizers [73]. 

To investigate the impact of different emulsifiers on the physicochemical properties of PSO emulsions, Han et al. systematically evaluated the effectiveness of gum arabic (GA), HI-CAP 100, Purity Gum 2000 (PG 2000), soluble soybean polysaccharide (SSPS), sodium caseinate (SC) and soy protein isolate (SPI) in stabilizing PSO emulsions. The results indicated that, except for emulsions prepared with SPI, the particle sizes of emulsions made with other emulsifiers primarily ranged from 0.1 to 10 μm, displaying a unimodal distribution. However, emulsions prepared with SPI and PG 2000 exhibited heterogeneous droplet size distribution and poor droplet uniformity. Further analysis revealed that PSO emulsions stabilized with 4% GA had the smallest droplet size, at only 0.678 μm, and demonstrated excellent stability [74]. Additionally, Chivero et al. investigated emulsions combining three different types of SSPS (SSPS-L, SSPS-M and SSPS-H) with three different oil phases (PSO, palm kernel oil and hexadecane). The study results showed that SSPS-L was most effective in reducing interfacial tension, particularly when hexadecane was used as the oil phase. Both SSPS-L and SSPS-M demonstrated superior emulsification performance by forming smaller droplets, which is attributed to their higher protein content, facilitating more efficient adsorption at the oil droplet interface. In contrast, SSPS-H exhibited poorer emulsification performance, resulting in larger droplet sizes and higher emulsion viscosity [52]. 

A significant increase in aqueous phase viscosity was identified as a key factor in hindering droplet movement, thereby enhancing emulsion stability. In further studies, researchers utilized SSPS as an emulsifier to prepare PSO emulsions, incorporating xanthan gum (XG) or guar gum (GG). As the concentration of gums increased from 0.03 wt.% to 0.3 wt.%, droplet size significantly decreased. Emulsions containing XG or GG displayed pseudoplastic behavior, indicating that viscosity decreased with increasing shear rate [75]. Moreover, these emulsions exhibited a lower creaming index, suggesting enhanced emulsion stability through increased viscosity of the continuous phase. Furthermore, chitosan, due to its positive charge, can adsorb onto the surface of soybean polysaccharides to form a bilayer emulsion. Yu et al. found that the addition of chitosan to PSO emulsions resulted in a shift of the Zeta potential from negative to positive values with increasing chitosan concentration, thereby enhancing emulsion stability. The optimal chitosan concentration was determined to be 0.4%, at which the Zeta potential reached 43 mV, with a droplet size of 1.185 μm. This concentration yielded emulsions with good physical stability and significant antioxidant activity [53].

The nanoemulsion system has garnered attention due to its nanoscale particle size, high stability and enhanced bioavailability [76]. Tunit et al. prepared microemulsions of moringa oil, PSO and mixed oils using Tween 80 as an emulsifier and the phase titration method, resulting in average particle + sizes of 159.33 nm, 183.86 nm and 263.43 nm, respectively. Additionally, the polydispersity index (PDI) and Zeta potential indicated good dispersibility and slightly negative stability of the microemulsions. The antioxidant activity of the mixed oil microemulsion (IC_50_ of 57.4 µg/mL) was significantly higher than that of the single oil microemulsions (moringa oil and PSO, with IC_50_ values of 140.5 µg/mL and 89.6 µg/mL, respectively), prove that the antioxidant activity of the mixed oil microemulsion is better than that of the single oil microemulsion. Suggesting that the mixed oil exhibits stronger 1,1-diphenyl-2-picrylhydrazyl (DPPH) radical scavenging capacity, potentially due to the synergistic effects of various fatty acids, such as oleic acid, eicosapentaenoic acid, cis-octadecenoic acid and palmitic acid [54,77]. Han et al. developed a PSO nanoemulsion using the Cremophor RH40-Span80 system, demonstrating that this nanoemulsion could enhance the bioavailability and stability of the oil. The nanoemulsion exhibited good stability across different storage temperatures (4 °C, 25 °C, 37 °C and 55 °C) and various NaCl concentrations (0, 2, 4, 6, 8 M). Furthermore, the MDA content, a lipid peroxidation product in the nanoemulsion, showed no significant changes over seven days, further confirming its stability. Additionally, intraperitoneal injection of P407 in mice indicated that lipid levels in the blood increased in both paraoxonase (PON) and lon protease (LON) treatment groups, suggesting enhanced intestinal absorption of P407 [56]. Research by Nguyen et al. demonstrated that emulsions prepared with 3 wt% octenyl succinic anhydride (OSA) starch maintained an average droplet diameter of 440 ± 3 nm over eight weeks of storage at 4 °C, with no significant changes observed. This finding indicates that OSA starch can effectively prevent droplet coalescence and flocculation at low temperatures. However, after four weeks of storage at 25 °C and 40 °C, the droplet diameters increased to 867 ± 40.3 nm and 891 ± 5.1 nm, respectively, indicating a tendency for droplet coalescence at higher temperatures [55]. High-pressure homogenization significantly reduced the particle size of the PSO nanoemulsion and enhanced its stability, achieving a minimum particle size of 293.87 ± 6.55 nm at 120 MPa. The nanoemulsion also improved the bioavailability of PSO [57].

Pickering emulsions are systems composed of two immiscible liquids stabilized by organic or inorganic solid particles. These solid particles, which possess specific sizes (micron or nanometer scale) and desired wettability, are considered ideal alternatives to conventional emulsifiers [78]. Studies have demonstrated that high internal phase emulsions stabilized by pea protein and PSO (PP-PSO HIPE) significantly influence the gel properties and conformation of myofibrillar protein (MP) gels. Results indicate that PP-PSO HIPE containing 4.0% (*w*/*v*) PP forms stable HIPE with small droplet size and good viscoelasticity. The addition of PP-PSO HIPEs (ranging from 5.0% to 15.0%) significantly enhances MP gel performance, with 10.0% PP-PSO HIPE exhibiting the highest gel strength and water-holding capacity. Furthermore, MP gels containing 10.0% PP-PSO HIPE display a higher proportion of bound water (PT22) and a lower proportion of free water (PT23). Additionally, these gels show a decrease in α-helix content and an increase in β-sheet structures, promoting superior gel characteristics. These findings suggest that PP-PSO HIPEs have potential applications in the development of reduced-fat meat products [58]. In addition, Li et al. explored the suitability of PSO Pickering emulsions stabilized by ovalbumin (OVA)-gum arabic (GA) polyelectrolyte complexes for spray drying and characterized the resulting powders. Compared to OVA, GA and their mixtures, OVA-GA complexes imparted greater stability to the emulsions. The viscosity of Pickering emulsions is highly sensitive to stabilizer concentration, with emulsions made from 2% OVA-GA complexes exhibiting acceptable viscosity and powder yield. In preventing oil leakage during spray drying, Pickering emulsions were more effective than OVA-stabilized emulsions, with the resulting powders containing up to 77.7% oil. Moreover, spray-dried Pickering emulsion powders demonstrated superior rehydration and flowability [59].

### 4.2. Oleogels

Many components that promote lipid oxidation, such as transition metals, enzymes and photosensitizers, are located in the aqueous phase [79]. The generation of secondary oxidation products not only produces unpleasant odors but can also lead to a decline in consumer interest in the product. Moreover, the formation of oxidation products significantly deteriorates the functional properties and quality of oil-based foods. Numerous studies have shown that introducing structuring agents into the dispersed phase through oleogel technology can promote internal crystallization of droplets, thereby forming “oleogel-structured emulsions” similar to nanostructured lipid carriers (NLC), which enhances the oxidative stability of PSO [80]. Prakansamut et al. used a blended oil composed of rice bran oil, camellia seed oil and PSO in a weight ratio of 80 g:10 g:10 g, with an additional 2 g of fish oil added per 100 g of the mixture. Subsequently, oleogels were prepared using different oleogelators, including monoglycerides, octadecanol and rice bran wax. The physicochemical properties and lipid quality indices of the selected mixed oil-based oleogels (OG) and their application in chocolate spreads (OG-CS) were analyzed. The hardness of all oleogel samples ranged from 0.12 to 1.25 N, and their spreadability ranged from 0.29 to 4.30 N·s. Monoglyceride oleogels exhibited significantly higher hardness and spreadability compared to octadecanol and rice bran wax oleogels. During 60 days of storage at 6–7 °C, the peroxide values of all OG-CS samples showed only a slight increase. These findings indicate that mixed oil-based oleogels have potential as structural fat substitutes in chocolate spreads [60]. Furthermore, Xu et al. investigated the effects of citral (CT) loading and fatty acid (FA) distribution on the physicochemical properties and in vitro digestion behavior of beeswax-based oleogels. Oleogels were prepared using various types of vegetable oils, such as coconut oil, palm oil, high-oleic peanut oil, safflower seed oil and PSO. The results demonstrated that oleogels containing high levels of unsaturated fatty acids (UFA), particularly those based on PSO, exhibited excellent oil-holding capacity (up to 99.03% ± 0.3%) and stable crystal network structures. When CT loading exceeded 10 wt%, the morphology of the oleogels might collapse. However, even at a 10 wt% CT loading level, PSO-based oleogels still exhibited high hardness (75.51 ± 6.91 g) and low free fatty acid (FFA) release rates (34.06% ± 1.48%), indicating superior gel strength and thermal stability. These findings suggest that UFA-rich oleogels, especially those based on PSO, can serve as healthy fat substitutes in functional foods while effectively encapsulating and releasing bioactive compounds such as citral [61].

### 4.3. Liposome

Liposomes, which are bilayer lipid vesicles surrounding an aqueous core, are widely used as delivery systems in the food, agricultural and pharmaceutical industries due to their ability to effectively encapsulate, protect and control the release of bioactive compounds [81]. Researchers have developed PSO nano-liposomes using a heating method and stabilized them by coating with one or two layers of biopolymer. The presence of biopolymer coatings was confirmed through FT-IR and TEM analyses. The resulting nano-liposomes exhibited particle sizes ranging from 200 to 502 nm and encapsulation efficiencies between 82% and 91%. After coating with chitosan and genipin, these nano-liposomes demonstrated excellent physical and oxidative stability. Under simulated gastric and intestinal conditions, the nano-liposomes showed good stability and effectively slowed the release of n-3 and n-6 polyunsaturated fatty acids. Further studies revealed that these nano-liposomes released less PSO in simulated gastric fluid but released more in simulated intestinal fluid, indicating their potential for controlled release to enhance the bioavailability of PSO in the gastrointestinal tract [62].

Additionally, Zheng et al. developed a NLC co-loaded with formononetin (FMN) and PSO using a melt-emulsification ultrasonic method. The prepared FMN-PSO-NLC had an average particle size of 117.5 ± 3.7 nm, a PDI of 0.240 ± 0.017 and a zeta potential of 36.7 ± 0.7 mV. The formation of NLCs was associated with changes in crystallinity and intermolecular interactions, and rheological studies indicated that they exhibited pseudoplastic flow behavior. The FMN-PSO-NLC demonstrated a good 24-h sustained release capability, with cumulative FMN release reaching 51.1% in simulated gastric fluid and increasing to 72.9% within 20 min in intestinal fluid. Antioxidant activity tests showed that FMN-PSO-NLC had a DPPH radical scavenging capacity IC50 value of 0.7 mg/mL, which was significantly better than that of PSO-NLC and FMN-SLN. Furthermore, when stored at 50 °C for 14 days, the peroxide value (PV) of FMN-PSO-NLC was lower than that of PSO-NLC, demonstrating superior oxidative stability, suggesting that the addition of formononetin significantly enhances the oxidative stability of oils [63].

### 4.4. Microcapsule

The success of microencapsulation systems relies on selecting appropriate wall materials and encapsulation techniques tailored to specific core materials. The choice of wall material is critical, as it significantly affects the properties of the resulting emulsions and microcapsules [82]. Ideally, the optimal wall material should exhibit excellent emulsifying properties, high water solubility, suitable drying characteristics and low viscosity. Due to the high ALA content in PSO, microcapsules of PSO have attracted considerable attention. Several studies have compared different microencapsulation methods [70] and types of wall materials to assess their effects on the characteristics of PSO microcapsules [83].

In investigating the influence of wall material types and concentrations on the properties of high-load PSO microcapsules, Han et al. used soluble soybean polysaccharides, gum arabic, Capsule starch, Pure-Cote 2000 and HI-CAP 100 as wall materials. They found that at a wall material concentration of 20%, microcapsules prepared with Pure-Cote 2000 and HI-CAP 100 exhibited lower surface oil content (3.05% and 3.31%, respectively) and higher encapsulation efficiency (93.86% and 93.33%, respectively), demonstrating superior encapsulation performance compared to other wall materials [64]. In another study, Yu et al. used gum arabic and β-cyclodextrin as wall materials to prepare microcapsules, which had a moisture content of 2.17%, bulk density of 0.43 g/cm^3^, an angle of repose of 36.27° and solubility of 74.65%, indicating good solubility properties. The microcapsules had a suitable particle size with an average diameter of 17.52 μm, which did not cause any discomfort during swallowing, and an encapsulation efficiency of 86.52%. The relative content of unsaturated fatty acids in the microencapsulated PSO was 70.85%, showing no significant difference from unencapsulated PSO, indicating that the spray drying method did not compromise the nutritional components of PSO. Additionally, the microcapsules underwent glass transition at 92.85 °C, suggesting that routine thermal treatments would not damage the surface structure of the microcapsules. With increased heat treatment duration, the peroxide value (POV) and thiobarbituric acid value (TBA) of the microcapsules rose significantly slower than those of the PSO, demonstrating better storage stability. These PSO microcapsules can be added to hot beverages, especially infant formula milk powder [65]. Xu et al. used flaxseed gum as the wall material to prepare PSO microcapsules without adding extra emulsifiers. Through single-factor experiments and orthogonal experiments, the optimal preparation conditions were determined (outlet temperature: 80 °C, inlet temperature: 180 °C, atomizer speed: 24,000 rpm, feed rate: 45.56 mL/min, wall-to-core material ratio: 3:2). Under these conditions, the microencapsulation efficiency of PSO reached 92.36%, with an oil content of 55.45% [66]. Furthermore, when Tween-80 and Span-80 were used as composite emulsifiers and solid corn syrup as a filler with PSO as the core material, the viscosity of the emulsion, particle size and encapsulation efficiency of the PSO microcapsules were measured to explore the effects of different wall materials, solid content and peristaltic pump speed on the preparation of PSO microcapsules. The results indicated that using sodium caseinate as the wall material with a mass fraction of 7.5% and a core-to-wall ratio of 3:1, and solid corn syrup as a filler with a mass fraction of 30%, and a granulation speed of 1200 r/min resulted in the highest encapsulation efficiency of 97.85% [67]. Stability studies showed that the microcapsule products exhibited good stability. When perilla protein powder and maltodextrin were used as wall materials, with PSO as the core material and Tween-80 and Span-80 as emulsifiers, optimal spray drying conditions were determined using orthogonal experiments. The results showed that under the conditions of 1% emulsifier addition (mass ratio of Tween-80 to Span-80 = 1:1), a core-to-wall ratio of 2:3 (cold-pressed PSO to composite wall material), a total solid content of 20%, inlet air temperature of 180 °C, feed rate of 4 L/h and rotation speed of 21,000 rpm, the encapsulation efficiency of the resulting microcapsule powder was 94.01%, with an oil content of 39.23% [68]. The microcapsule powder exhibited good sensory properties and strong storage stability. When gelatin and gum arabic were used as wall materials, optimal conditions for preparing PSO microcapsules were determined by adjusting factors such as wall material concentration, pH, core-to-wall ratio and stirring speed. A mass ratio of 1:1 for gelatin to gum arabic, a core-to-wall ratio of 1:1, a wall material mass fraction of 1%, a pH value of 3.9 and a stirring speed of 300 rpm were found to be optimal. Observations using optical microscopy and scanning electron microscopy revealed that the microcapsules prepared under these conditions had complete structures, uniform shapes and homogeneous dispersion [69].

In comparisons of microencapsulation methods, microcapsules prepared by spray drying achieved an encapsulation efficiency (EE) of up to 98.86%, whereas those prepared by freeze-drying had an EE of 76.24%. These findings indicate that spray drying yields significantly higher encapsulation efficiency than freeze-drying, effectively reducing the exposure of PSO to the external environment and substantially extending its shelf life. Microcapsules produced via spray drying feature smooth surfaces, regular spherical shapes, fine textures, good flowability and easy solubility, thereby greatly enhancing the potential applications of PSO in functional foods. Moreover, the spray drying process requires simple equipment, is flexible in operation, has low cost and yields high output. In contrast, microcapsules produced through freeze-drying tend to be loose, porous, irregularly shaped, exhibit poor flowability and have higher moisture content [70]. Further research has demonstrated that optimizing emulsifier ratios, homogenization conditions and process parameters using response surface methodology can significantly enhance the stability of the PSO microcapsule emulsion. The optimal emulsion stability was achieved with a composite emulsifier ratio of Tween 80 to Span 60 at 7:3 (HLB value of 11.91). When using freeze-drying for microencapsulation, adding 1% ice-structuring proteins (ISPs) as cryoprotectants increased the encapsulation efficiency to 73.7%, a 6.6% improvement compared to freeze-drying without ISPs. The resulting freeze-dried microcapsule powder appeared pale yellow, with a moisture content of 3.3%, bulk density of 0.35 g/mL, flowability score of 39 and solubility of 73.37%. In in vitro simulated digestion experiments, the release rate of freeze-dried microcapsules containing ISPs reached 90.97% within 9 h. Additionally, under environmental conditions such as oxygen, temperature and light exposure, freeze-dried microcapsules exhibited superior stability compared to the spray-dried group, especially after adding ISPs; in an aerobic environment, the encapsulation efficiency decreased by only 25.99% over 28 days. Accelerated oxidation tests showed that the peroxide value of freeze-dried microcapsules with ISPs was 10.5 mmol/kg after 14 days of storage, significantly enhancing the oxidative stability and shelf life of PSO [84]. Furthermore, storage conditions had a substantial impact on the stability of the microcapsules, specifically regarding the total phenolic content (TPC), acid value, peroxide value and antioxidant capacity of both free PSO and encapsulated PSO. During storage, the samples exhibited no noticeable changes in color or odor, and no precipitation or phase separation occurred. While the acid value of PSO remained relatively stable, the peroxide value of PSO stored at 40 °C increased significantly, whereas the peroxide value of PSO showed no such changes. In addition, TPC and antioxidant capacity were not significantly affected under various storage conditions. The study concluded that PSO demonstrated higher stability against oxidation compared to free PSO [85]. PSO microcapsules were successfully prepared using vacuum drying, and their morphology was observed through optical microscopy and scanning electron microscopy. Single-factor experiments and response surface optimization determined the optimal conditions to be a core-to-wall mass ratio of 1.5:1, coagulation time of 60 min, temperature of 40 °C and pH of 3.8. Under these conditions, the theoretical encapsulation efficiency reached 90.1%, with an actual verified efficiency of 88.3%. The optimized PSO microcapsules exhibited high stability at room temperature, and their thermal stability was significantly superior to untreated PSO [71].

Overall, these studies primarily focus on the optimization of processing techniques and the characteristics of microcapsules; however, there has been limited exploration of the stability of the oil and emulsion properties prior to drying [83].

## 5. Conclusions and Perspectives

PSO has high nutritional and medicinal value and offers promising commercial prospects. The findings of this review suggest that PSO exhibits a wide range of bioactivities, including antioxidant, anti-inflammatory, lipid-lowering, hypoglycemic, neuroprotective and immune-enhancing effects. However, the underlying mechanisms of these bioactivities are not well understood, and the specific molecular mechanisms have not been fully elucidated. Further in-depth mechanistic studies are necessary to understand how PSO exerts its effects at the molecular level, which could provide a theoretical basis for its clinical applications. Most research on PSO has been conducted using in vitro experiments or animal models. Although these studies provide preliminary evidence, there is a lack of large-scale human clinical trials to validate its health benefits. Additionally, research on the effects of PSO on gut microbiota is limited, with current studies primarily focusing on changes in microbial abundance rather than elucidating the underlying mechanisms. These studies only suggest a positive correlation without establishing causation. Future research should employ multi-omics approaches to explore how PSO influences bioactivity. PSO is rich in unsaturated fatty acids, making it susceptible to oxidation, which limits its application in food and pharmaceuticals. Although various encapsulation techniques have been developed to enhance its stability and bioavailability, the efficacy of these techniques requires further validation. Moreover, different encapsulation methods may vary in their ability to improve the bioavailability of PSO, and there is a lack of systematic comparative studies in this area. Targeted research on these techniques could be beneficial. Research on the multifunctionality and synergistic effects of PSO is insufficient. Some studies have shown that combining PSO with other functional components, such as other plant oils or antioxidants, can lead to enhanced effects. However, the mechanisms underlying these synergistic effects and the optimal combinations have not been adequately studied. Systematic research on the interactions between the multifunctional properties of PSO and other nutritional components will aid in the development of more effective nutritional and health products. In conclusion, while the potential health benefits of PSO are promising, further research is needed to understand its mechanisms of action, validate its effects through clinical trials and optimize its applications through advanced encapsulation techniques and synergistic combinations with other bioactive compounds.

## Figures and Tables

**Table 1:** Health benefits of PSO reported in literatures.

Health Benefits	Test Subjects	Types	Effects	Reference
Antioxidant Activity	O^2−^ and NaNO_2−_	In vitro	The IC_50_ values are 2.49 mg/mL and 2.51 mg/mL.	[11]
	DPPH	In vitro	The scavenging ability is 73.14%.	[12]
	Murine embryonic fibroblasts	In vitro	ROS ↓	[13]
	TNF-α-induced A549 lung adenocarcinoma cells	In vitro	Oxidative stress ↓	[14]
	Ethanol-induced oxidative damage in mice	In vivo	MDA and PC ↓ GSH and SOD ↑	[15]
Anti-inflammatory activity	A549 cells	In vitro	IL-1β, IL-6, IL-8, TNF-α, COX-2 and LTB4 ↓	[16]
	(EPP)-induced ear swelling in rats	In vivo	Twisting response ↓ Ear response ↓	[17]
Hypolipidemic activity	Hyperlipidemic mice	In vivo	TC, TG, LDL-C and MDA ↓ HDL-C, SOD and CAT ↑	[18,19,20]
Hypoglycemic activity	Spontaneous diabetic KKAy mice	In vivo	ALT and AST↑ Glucose-6-phosphate dehydrogenase, TG and TC↓	[21]
	Type 2 diabetic KKAy mice	In vivo	Insulin levels ↑ Blood glucose levels ↓	[22]
Neuroprotective activity	Chronic cerebral hypoperfusion rats	In vivo	Escape latency and TChE ↓ TChE ↓	[23]
	Weanling rats	In vivo	Escape latency↓ Platform crossing frequency↑	[24]
	ApoE KO mice	In vivo	Dark neurons ↓ Neuronal apoptosis ↓	[25]
Immunoregulatory activity	layer chicks	In vivo	Nonspecific IgG ↑	[26]
	OVA-sensitized mice	In vivo	Nonspecific IgE ↑ Nonspecific IgA ↓	[27]
	Rats	In vivo	Spleen index, thymus index and phagocytic index ↑ HC_50_ ↑	[28]
Antibacterial activity	*Escherichia coli* and *Bacillus subtilis*	In vitro	The inhibition rates were 53.3–101.7% and 108.3–136.7%, respectively.	[29]

Abbreviations: IC_50_, half maximal inhibitory concentration; DPPH, 2,2-diphenyl-1-picrylhydrazyl; ROS, reactive oxygen species; TNF-α, tumor necrosis factor-alpha; A549, human lung adenocarcinoma cells; MDA, malondialdehyde; PC, protein carbonyl; GSH, glutathione; SOD, superoxide dismutase; COX-2, cyclooxygenase-2; LTB4, leukotriene B4; TC, total cholesterol; TG, triglycerides; LDL-C, low-density lipoprotein cholesterol; HDL-C, high-density lipoprotein cholesterol; CAT, catalase; ALT, alanine aminotransferase; AST, aspartate aminotransferase; TChE, total cholinesterase; IgG, immunoglobulin G; IgE, immunoglobulin E; IgA, immunoglobulin A; HC_50_, hemolytic complement 50% endpoint; ApoE KO, apolipoprotein E knockout; and OVA, ovalbumin.

**Table 2:** The encapsulation strategies of PSO.

Encapsulation Type	Condition	Physicochemical Property	Reference
Emulsions	Emulsifier: GA (mass fraction: 4%)	Particle size: 0.678 ± 0.006 μm Emulsion instability index: 0.131 Slope values: 0.0015%/s	[33]
	Emulsifier: HI-CAP 100 (mass fraction: 16%)	Particle size: 0.709 ± 0.045 μm Emulsion instability index < 0.2 Slope values < 0.0025%/s	
	Emulsifier: PG 2000 (mass fraction: 4%)	Particle size: 0.766 ± 0.014 μm Emulsion instability index < 0.2 Slope values < 0.0025%/s	
	Emulsifier: SSPS (mass fraction: 16%)	Particle size: 0.735 ± 0.004 μm Emulsion instability index < 0.2 Slope values < 0.0025%/s	
	Emulsifier: SPI (mass fraction: 1%)	Particle size: 2.075 ± 0.095 μm Emulsion instability index < 0.2 Slope values < 0.0025%/s	
	Emulsifier: SC (mass fraction: 4%)	Particle size: 0.742 ± 0.003 μm	
	Emulsifier: SSPS-L (mass fraction: 6%)	Particle size: 0.07 mm	[52]
	Emulsifier: SSPS-M (mass fraction: 6%)	Particle size: 0.08 mm	
	Emulsifier: SSPS-H (mass fraction: 6%)	Particle size: 0.02 mm	
	Emulsifiers: Soybean polysaccharides (core-to-wall ratio 2:1) and Chitosan (0.4%)	Particle size: 1.185 μm Zeta potential: +43 mV	[53]
	Emulsifier: Tween 80 Oil phase: moringa seed oil and perilla seed oil	Particle size: 159.33–263.43 nm PDI: 0.341 ± 0.002–0.350 ± 0.018 Zeta potential: −7.636 ± 0.525–−6.61 ± 0.17 mV The rheological property: pseudoplastic flow behavior	[54]
	Emulsifier: OSA-Starch (mass fraction 3%)	Particle size: 440 nm (4 °C) and 867–891 nm (25–40 °C)	[55]
	Emulsifier: Cremophor RH40-Span80 (3:1) (mass fraction: 30%) Oil-to-water ratio: 3:7	Particle size: 293.87 nm Zeta potential: −43.2 mV	[56]
	Emulsifiers: SPI (4% *w*/*v*) and Phosphatidylcholine 0.4% (*w*/*v*) Homogenization Pressure: 120 MPa	Particle size: 293.87 ± 6.55 nm PDI < 0.25	[57]
	Emulsifier: Pea Protein (4.0% *w*/*w*) Oil-to-Water Ratio: 3:1	Particle size: 23.39 μm Zeta potential: −29.00 mV	[58]
	Emulsifier: Ovalbumin (OVA)–Gum Arabic (GA) (mass fraction: 2%)	Particle size: 3176.33–4336.00 nm Zeta potential: −17.99–19.91 mV Oil content: 77.7% Pseudoplastic behavior	[59]
Oleogels	Oil ratio: 80 g:10 g:10 g (rice bran oil: camellia seed oil: perilla seed oil) Gelators: monoglycerides, polyols and rice bran wax	Hardness: 1.25 ± 0.16 N, 0.75 ± 0.06 N and 0.12 ± 0.03 N Spreadability: 4.30 ± 1.0 N·s, 2.49 ± 0.29 N·s and 0.29 ± 0.14 N·s Melting point: 53.50 °C, 54.00 °C and 40.00 °C	[60]
	Gelling agent: Beeswax Citral (CT) < 10 wt%	Oil-Holding Capacity (OHC) value: 99.03% Hardness: 192.59 g Melting onset temperature (Tonset): 30.81 °C Melting peak temperature (Tpeak): 44.48 °C FFA release rate: 34.06%	[61]
Liposome	Liposome: L-α-lecithin Coating materials: chitosan, poly-L-lysine and sodium alginate Crosslinking agent: genipin	Particle size: 200–502 nm Zeta potential: +41.9 mV Encapsulation efficiency: 82–91% Fatty acid release: 20–80%	[62]
	Surfactants: Tween 80 and 1,2-Propanediol Solid Lipid: Glyceryl Distearate Liquid Lipid: PSO	Particle size: 117.5 ± 3.7 nm PDI: 0.240 ± 0.017 Zeta potential: 36.7 ± 0.7 mV DPPH radical scavenging ability: IC_50_ of 0.7 mg/mL	[63]
Microcapsule	Wall Materials: Pure-Cote 2000 and HI-CAP 100 (mass fraction:20%)	Particle Size: 109.4 ± 5.5 μm and 110.3 ± 6.8 μm Surface Oil Content: 3.05% and 3.31%, respectively Encapsulation Rate: 93.86% and 93.33%, respectively	[64]
	Wall materials: Arabic gum and β-cyclodextrin (β-CD) (1:2) Core materials: Mixture of monoesters and sucrose esters (6:4) and PSO	Particle size: 17.52 ± 1.86 μm Encapsulation efficiency: 86.52% Bulk density: 0.43 g/cm^3^ Moisture content: 2.17 ± 0.11% Solubility: 74.65 ± 0.34% Contact angle: 36.27 ± 0.17°	[65]
	Wall material: Flaxseed gum Core-to-wall ratio: 3:2	Encapsulation efficiency: 97.9% Microencapsulation efficiency: 92.36% Oil content: 55.45%	[66]
	Wall material: Sodium caseinate (mass fraction: 7.5%) Core-to-wall ratio: 3:1 Filler: Solid corn syrup (mass fraction: 30%)	Encapsulation efficiency: 97.85%	[67]
	Emulsifiers: Tween-80 and Span-80 (Mass ratio 1:1, Mass fraction 1%) Wall Materials: Perilla Protein Powder and Maltodextrin (Mass ratio 3:1) Core-to-Wall Ratio: 2:3	Encapsulation Rate: 94.01% Oil Content: 39.23%	[68]
	Gelatin: Arabic gum (1:1 ratio, 1% mass fraction) Core-to-wall ratio: 1:1	Structure intact Uniform morphology Even dispersion	[69]
	Wall Materials: Octenyl succinic anhydride modified starch Solid Content: 30% Core-to-Wall Ratio: 1: 4	Microencapsulation Efficiency: 98.86% and 76.24% (Spray Drying and Freeze-Drying)	[70]
	Wall Material: Gelatin Core-to-Wall Ratio: 1.5:1	Encapsulation Rate: 89.4% Particle size: 20–40 μm	[71]

Abbreviations: GA, gum arabic; PG, Purity Gum; SSPS, soluble soybean polysaccharide; SC, sodium caseinate; SPI, soy protein isolate; SSPS-L, SSPS+ PSO; SSPS-M, SSPS +palm kernel oil; SSPS-H, SSPS +hexadecane; OSA, octenyl succinic anhydride.

## Data Availability

No new data were created or analyzed in this study. Data sharing is not applicable to this article.

## References

[1] Yang X., Li Y., Ma J., Wu F., Wang L., Sun L., Zhang P., Wang W., Xu J. (2022). Comparative physiological and soil microbial community structural analysis revealed that selenium alleviates cadmium stress in *Perilla frutescens*. Front. Plant Sci..

[2] Fuyuno Y., Uchi H., Yasumatsu M., Morino-Koga S., Tanaka Y., Mitoma C., Furue M. (2018). Perillaldehyde Inhibits AHR Signaling and Activates NRF2 Antioxidant Pathway in Human Keratinocytes. Oxidative Med. Cell. Longev..

[3] Tian J., Zeng X., Zhang S., Wang Y., Zhang P., Lue A., Peng X. (2014). Regional variation in components and antioxidant and antifungal activities of *Perilla frutescens* essential oils in China. Ind. Crops Prod..

[4] Lee K.-R., Kim K.-H., Kim J.B., Hong S.-B., Jeon I., Kim H.U., Lee M.H., Kim J.K. (2019). High accumulation of γ-linolenic acid and Stearidonic acid in transgenic Perilla (*Perilla frutescens* var. *frutescens*) seeds. BMC Plant Biol..

[5] Asif M. (2011). Health effects of omega-3,6,9 fatty acids: Perilla frutescens is a good example of plant oils. Orient. Pharm. Exp. Med..

[6] Liang X., Wang X., Chen D., Song N. (2022). The Research and Application Status of Perilla Seed Oil. China J. Chin. Med..

[7] Jae Kwang K., Soo-Yun P., Jong-Kuk N., Eun Soo S., Chang Yeon Y. (2012). Metabolite profiling based on lipophilic compounds for quality assessment of perilla (*Perilla frutescens*) cultivars. J. Agric. Food Chem..

[8] Seonyeong W., Hyunsuk H., Sukhoo Y., Eunok C. (2010). Temperature dependence of autoxidation of perilla oil and tocopherol degradation. J. Food Sci..

[9] Tingting Z., Seung In H., Junsoo L., Jeom-Sig L., In-Hwan K. (2012). Impact of roasting on the chemical composition and oxidative stability of perilla oil. J. Food Sci..

[10] Timilsena Y.P., Wang B., Adhikari R., Adhikari B. (2017). Advances in microencapsulation of polyunsaturated fatty acids (PUFAs)-rich plant oils using complex coacervation: A review. Food Hydrocoll..

[11] Cao X., Yang H. (2018). Optimization of Ultrasonic-assisted Extraction for Perilla frutescens Seed Oilby Response Surface Methodology and lts Antioxidant Activity. China Condiment.

[12] Huang J., Xu Y., Chen C., Song Z., Chang M., Wang X., Wang X. (2024). Effect of infrared roasting of perilla seeds on the content of bioactive components and antioxidant capacity in oil. J. Am. Oil Chem. Soc..

[13] Lee S., Lee Y.-J., Sung J.-S., Shin H.-S. (2015). Influence of roasting conditions on the chemical properties and antioxidant activity of perilla oils. J. Korean Soc. Appl. Biol. Chem..

[14] Chen X., Hu B., Tang X., Zhang B. (2024). Effects of Perilla seed oil on intestinal microorganisms and antioxidant capacity in mice. Food Ferment. Ind..

[15] Zhang Y., Sun H., Liu Z., Zhou H. (2015). Antioxidant Effect of Perilla Oil on Ethanol-Induced Oxidative Stress in Mice. Food Sci..

[16] Zhang H., Tian Y., Guan J., Xie Q., Zhao Y. (2021). The anti-tussive, anti-inflammatory effects and sub-chronic toxicological evaluation of perilla seed oil. J. Sci. Food Agric..

[17] Paradee N., Koonyosying P., Kusirisin W., Janthip R., Kanjanapothi D., Pattanapanyasat K., Srichairatanakool S. (2021). Analgesic, anti-inflammatory and anti-ulcer properties of Thai *Perilla frutescence* fruit oil in animals. Biosci. Rep..

[18] Li Y., Zhang Y. (2008). The hypolipidemic effect of perilla seed oil. J. Environ. Health.

[19] Wang W. (2022). Study on Hypidemic Effect of Perilla Seed Oil and Preparation of Micropsule. Master’s Thesis.

[20] Li F., Zhang M., Wang X. (2023). Feasibility of Safflower Seed Oil and Basil Seed Oil Mixture in Intervention of Hyperlipidemia. China J. Chin. Med..

[21] Wang F., Zhu H., Hu M., Wang J., Xia H., Yang X., Yang L., Sun G. (2018). Perilla Oil Supplementation Improves Hypertriglyceridemia and Gut Dysbiosis in Diabetic KKAy Mice. Mol. Nutr. Food Res..

[22] Wang J., He Y., Yu D., Jin L., Gong X., Zhang B. (2020). Perilla oil regulates intestinal microbiota and alleviates insulin resistance through the PI3K/AKT signaling pathway in type-2 diabetic KKAy mice. Food Chem. Toxicol..

[23] Wu J., Li H., Yang Y. (2012). Effect of perilla oil on chronic hypoperfusion of the brain induced cognitive injury in rats. West China J. Pharm. Sci..

[24] Chen L., Wang L., Guo Y., Xiao N., Tao X., Liu Z., Liu D., Liu L. (2011). Effect of walnut oil, perilla oil,α-linolenic acid and linoleic acidsupplementation on rats spatial learning and memory ability. China Oils Fats.

[25] Seong J., Song Y.O. (2012). Perilla oil rich in α-linolenic acid inhibits neuronal apoptosis and the expression of inflammation-mediator protein in apoE KO mice. Biocatal. Agric. Biotechnol..

[26] Cheng H., Tian C., Cai F., Pan X., Li K., Zhang J., Hong Z. (2020). Effects of perilla seed oil on the growth and development, immunefunction and PUFAs composition of young laying chickens. Anim. Husb. Vet. Med..

[27] Chang H.-H., Chen C.-S., Lin J.-Y. (2009). Dietary perilla oil lowers serum lipids and ovalbumin-specific IgG1, but increases total IgE levels in ovalbumin-challenged mice. Food Chem. Toxicol..

[28] Xu L., Pan W., Cui J. (2021). Response surface methodology optimization of water extraction of perillaseed oil and its effect on immune function of exercise rats. Cereals Oils.

[29] Wang S., Zhang L., Li P., Wang X., Zhang Q. (2017). Antibacterial Effects of Perilla Seed Oil Against *Escherichia coli* and *Bacillus subtilis*. Food Nutr. China.

[30] Jia X., Zhang Q., Liu H., Wang X., Zeng Y., Zhong G. (2022). Effect of infrared preheating treatment on quality and antioxidant activityof pressed perilla seed oil. China Oils Fats.

[31] Wu R., Ma F., Zhang L., Li P., Zhang W., Zhang Q., Li G. (2015). Simultaneous Determination of 11 Phenolic Compounds in Perilla oil by High Performance Liquid ChromatographyTandem Mass Spectrometry. Chin. J. Anal. Chem..

[32] Wu C. (2012). Studies on Anti-Aging Effect and Mechanism of Perilla Oil. Master’s Thesis.

[33] Xu J., Zhang J., Lin H., Zhang J., Zhou R., Wu X., Niu Y., Zhang J. (2023). Preparation of oral nanoparticles of Perillae Fructus oil and prevention application of cold stress in mice. Food Sci. Nutr..

[34] Wang X., Cao Y., Chen S., Lin J., Bian J., Huang D. (2021). Anti-inflammation activity of flavones and their structure–activity relationship. J. Agric. Food Chem..

[35] Chang H.-H., Chen C.-S., Lin J.-Y. (2012). Protective effect of dietary perilla oil on allergic inflammation in asthmatic mice. Eur. J. Lipid Sci. Technol..

[36] Huang Z.-R., Chen M., Guo W.-L., Li T.-T., Liu B., Bai W.-D., Ai L.-Z., Rao P.-F., Ni L., Lv X.-C. (2020). Monascus purpureus-fermented common buckwheat protects against dyslipidemia and non-alcoholic fatty liver disease through the regulation of liver metabolome and intestinal microbiome. Food Res. Int..

[37] Mohamed S. (2014). Functional foods against metabolic syndrome (obesity, diabetes, hypertension and dyslipidemia) and cardiovasular disease. Trends Food Sci. Technol..

[38] Shen S., Chang J., Chai Y., Li J., Chu M., Li L. (2020). Hypolipidemic effect of different proportions of safflower seed oil and perilla seed oil. China Oils Fats.

[39] Ma H., Zhang Y. (2022). Security and hypolipidemic effect of perilla seed oil and linseed oil in combination. Mod. Prev. Med..

[40] Yu D., Ma C., Song L., Long X., Geng Y. (2014). Lipid-lowering effect of perilla seed oil in combination with safflower seed oil. China Oils Fats.

[41] Sun K., Li X., Ma T. (2024). Research progress on the chemical composition and functional activities of perilla seed oil. Grain Oil Feed Technol..

[42] Jang J.-Y., Kim T.-S., Cai J., Kim J., Kim Y., Shin K., Kim K.-S., Lee S.-P., Kang M.-H., Choi E.-K. (2014). Perilla oil improves blood flow through inhibition of platelet aggregation and thrombus formation. Lab. Anim. Res..

[43] Cha Y., Jang J.Y., Ban Y.-H., Guo H., Shin K., Kim T.-S., Lee S.-P., Choi J., An E.-S., Seo D.-W. (2016). Anti-atherosclerotic effects of perilla oil in rabbits fed a high-cholesterol diet. Lab. Anim. Res..

[44] Sommer F., Bäckhed F. (2013). The gut microbiota—Masters of host development and physiology. Nat. Rev. Microbiol..

[45] Tremaroli V., Bäckhed F. (2012). Functional interactions between the gut microbiota and host metabolism. Nature.

[46] Zhang X., Wang H., Yin P., Fan H., Sun L., Liu Y. (2017). Flaxseed oil ameliorates alcoholic liver disease via anti-inflammation and modulating gut microbiota in mice. Lipids Health Dis..

[47] Lee W., Tung Y., Wu C., Tu P., Yen G. (2018). Camellia Oil (*Camellia oleifera* Abel.) Modifies the Composition of Gut Microbiota and Alleviates Acetic Acid-Induced Colitis in Rats. J. Agric. Food Chem..

[48] Miao F., Shan C., Ma T., Geng S., Ning D. (2021). Walnut oil alleviates DSS-induced colitis in mice by inhibiting NLRP3 inflammasome activation and regulating gut microbiota. Microb. Pathog..

[49] Martinez N., Prieto I., Hidalgo M., Belen Segarra A., Martinez-Rodriguez A.M., Cobo A., Ramirez M., Galvez A., Martinez-Canamero M. (2019). Refined versus Extra Virgin Olive Oil High-Fat Diet Impact on Intestinal Microbiota of Mice and Its Relation to Different Physiological Variables. Microorganisms.

[50] Farras M., Martinez-Gili L., Portune K., Arranz S., Frost G., Tondo M., Blanco-Vaca F. (2020). Modulation of the Gut Microbiota by Olive Oil Phenolic Compounds: Implications for Lipid Metabolism, Immune System, and Obesity. Nutrients.

[51] Aldamarany W.A.S., Taocui H., Deng L., Mei H., Yi Z., Zhong G. (2023). Perilla, sunflower, and tea seed oils as potential dietary supplements with anti-obesity effects by modulating the gut microbiota composition in mice fed a high-fat diet. Eur. J. Nutr..

[52] Chivero P., Gohtani S., Yoshii H., Nakamura A. (2014). Physical properties of oil-in-water emulsions as a function of oil and soy soluble polysaccharide types. Food Hydrocoll..

[53] Yu Y., Gao Y., Zhu Q., Mou D., Duan S., Hou Z. (2019). Preparation and Physicochemical Stability of Perilla Seed Oil Double-Layer Emulsions. Food Sci..

[54] Tunit P., Kietinun S., Sriyakul K., Tungsukruthai P., Chittasupho C. (2020). Enhancement of Antioxidant Activity by the Combination of Moringa oleifera and Perilla frutescens Seed Oils in Microemulsion. Key Eng. Mater..

[55] Nguyen M.-T., Shin J.-A., Lee K.-T. (2023). Oxidation stability of oil-in-water emulsion prepared from perilla seed oil and soy sauce with high salt concentration using OSA-starch. Food Sci. Biotechnol..

[56] Han B., Yu B., Liu L., Xiu Y., Wang H. (2019). Experimental investigation of the strong stability, antibacterial and anti-inflammatory effect and high bioabsorbability of a perilla oil or linseed oil nanoemulsion system. RSC Adv..

[57] Hu M., Xie F., Zhang S., Qi B., Li Y. (2021). Effect of nanoemulsion particle size on the bioavailability and bioactivity of perilla oil in rats. J. Food Sci..

[58] Li B., Wang Y., Wang S., Chen S., Yang C., Liu L., Bi S., Zhou Y., Zhu Q. (2024). Perilla seed oil high internal phase emulsion improve the gel properties of myofibrillar protein. Food Chem. X.

[59] Li K.-Y., Zhou Y., Huang G.-Q., Li X.-D., Xiao J.-X. (2022). Preparation of powdered oil by spray drying the Pickering emulsion stabilized by ovalbumin–gum Arabic polyelectrolyte complex. Food Chem..

[60] Prakansamut N., Adulpadungsak K., Sonwai S., Aryusuk K., Lilitchan S. (2024). Application of functional oil blend-based oleogels as novel structured oil alternatives in chocolate spread. LWT Food Sci. Technol..

[61] Xu B., Lin X., Zhao Y., Yin C., Cheng Y., Li X., Li Y. (2024). The effect of citral loading and fatty acid distribution on the oleogels: Physicochemical properties and in vitro digestion. Food Chem..

[62] Zamani-Ghaleshahi A., Rajabzadeh G., Ezzatpanah H., Ghavami M. (2020). Biopolymer coated nanoliposome as enhanced carrier system of perilla oil. Food Biophys..

[63] Zheng L., Xu H., Zhang H., Shi C., Zhou W., Zhang X. (2023). Nanostructured lipid carrier as a strategy for encapsulation of formononetin and perilla seed oil: In vitro characterization and stability studies. Food Biosci..

[64] Han L., Hou Z., Wen J., Wu Y., Qian Y., Gong S. (2016). Preparation and evaluation of high-loaded perilla seed oil by microencapsulation with different wall materials. Sci. Technol. Food Ind..

[65] Yu J., Zhang W., Wang H., Sun H., Li C. (2023). Preparation, Physicochemical Indexes and Application Potential of Perilla Seed Oil Microcapsule. Chin. Agric. Sci. Bull..

[66] Xu J., Xiao J., Chen Y., Zhang W., Wang J., Wang P., Mi L., You P. (2013). Research on Microencapsulation of Perilla Seed Oil Produced by Spray-drying Method. China Condiment.

[67] Zhu X., Li J., Pan Y., Wang N. (2021). The Research of Spray Granulation Technolgy and Stability of Perilla Seed Oil Microcapsules. Beverage Ind..

[68] Li X., Han F., Zhang Z., Li H., Chen T. (2018). Preparation of perilla seed oil microcapsules. Food Sci. Technol..

[69] Chang D., Zhang T., Ma R., Zheng C., Fang C. (2019). Study on preparation of perilla oil microcapsules by complex coacervation followed by freeze drying. J. Shaanxi Univ. Sci. Technol..

[70] Chen L., Li R., Jiang Z., Tan J. (2013). Comparative study on properties of the microencapsule of perilla oilentrapped with different microencapsulation methods. Sci. Technol. Food Ind..

[71] Jiang A. (2022). Preparation and Application of Single-Walled Mierocapsules. Master’s Thesis.

[72] Wang Y., Ai C., Wang H., Chen C., Teng H., Xiao J., Chen L. (2023). Emulsion and its application in the food field: An update review. eFood.

[73] Pang M., Zheng D., Jia P., Cao L. (2022). Novel Water-in-Oil Emulsions for Co-Loading Sialic Acid and Chitosan: Formulation, Characterization, and Stability Evaluation. Foods.

[74] Han L., Hou Z., Wen J., Wu Y., Qian Y., Gong S. (2016). Preparation and stability evaluation of perilla seed oil emulsions. Food Ferment. Ind..

[75] Chivero P., Gohtani S., Yoshii H., Nakamura A. (2015). Effect of xanthan and guar gums on the formation and stability of soy soluble polysaccharide oil-in-water emulsions. Food Res. Int..

[76] Walker R., Decker E.A., McClements D.J. (2015). Development of food-grade nanoemulsions and emulsions for delivery of omega-3 fatty acids: Opportunities and obstacles in the food industry. Food Funct..

[77] Tunit P., Chittasupho C., Sriyakul K., Tungsuruthai P., Chakkavittumrong P., Na-Bangchang K., Kietinun S. (2022). Emulgels Containing *Perilla frutescens* Seed Oil, *Moringa oleifera* Seed Oil, and Mixed Seed Oil: Microemulsion and Safety Assessment. Polymers.

[78] Brito de Carvalho-Guimaraes F., Correa K.L., de Souza T.P., Rodriguez Amado J.R., Ribeiro-Costa R.M., Silva-Junior J.O.C. (2022). A Review of Pickering Emulsions: Perspectives and Applications. Pharmaceuticals.

[79] McClements D.J., Decker E. (2018). Interfacial Antioxidants: A Review of Natural and Synthetic Emulsifiers and Coemulsifiers That Can Inhibit Lipid Oxidation. J. Agric. Food Chem..

[80] Wei F., Miao J., Tan H., Feng R., Zheng Q., Cao Y., Lan Y. (2021). Oleogel-structured emulsion for enhanced oxidative stability of perilla oil: Influence of crystal morphology and cooling temperature. LWT Food Sci. Technol..

[81] Rudzińska M., Grygier A., Knight G., Kmiecik D. (2024). Liposomes as Carriers of Bioactive Compounds in Human Nutrition. Foods.

[82] Gharsallaoui A., Roudaut G., Chambin O., Voilley A., Saurel R. (2007). Applications of spray-drying in microencapsulation of food ingredients: An overview. Food Res. Int..

[83] Zhang C., Zhou W., Xiang J., Chen H., Quek S.Y. (2022). Fabrication, characterization, and oxidative stability of perilla seed oil emulsions and microcapsules stabilized by protein and polysaccharides. J. Food Process. Preserv..

[84] Li Y. (2020). Study on the Effect of Ice Structuring Protein on Freeze-Dried Perilla Oil Microcapsule Powder. Master’s Thesis.

[85] Chaiyasut C., Sivamaruthi B.S., Kesika P., Sirilun S., Makhamrueang N., Peerajan S. (2019). Evaluation of Pharmacological Stability of Perilla Oil and Perilla Oil Capsule. Asian J. Pharm. Clin. Res..

